# “It also taught me a lot about myself”: A qualitative exploration of how men understand eating disorder recovery

**DOI:** 10.1186/s40337-020-0279-6

**Published:** 2020-02-03

**Authors:** Ranidu S. Lewke-Bandara, Priyanka Thapliyal, Janet Conti, Phillipa Hay

**Affiliations:** 1 School of Medicine, Western Sydney University, Sydney, Australia; 2 Western Sydney University, Sydney, Australia; 3 School of Social Sciences and Psychology, Western Sydney University, Sydney, Australia; 4 Translational Health Research Institute, School of Medicine, Western Sydney University, Locked Bag 1797, Penrith, NSW 2751 Australia

**Keywords:** Eating disorder, Anorexia Nervosa, recovery, men, journey, interpersonal relationships

## Abstract

**Background:**

Eating Disorders (ED) are relatively common in the general population. However, perceived as “female disorders”, EDs in men are often overlooked. Although recovery is often seen as the ideal end goal of ED, there is no single universal definition of recovery. Recovery can be defined in terms of: physical changes, behavioural changes, psychological (cognitive and emotional) and improved quality of life. There is very little research exploring how people with ED define/ understand what recovery is and there is even less research involving men with ED. Therefore, the aim of this study was to explore recovery from men’s perspectives.

**Methods:**

In this qualitative study, eight men from Australia, New Zealand and the United States participated in a semi-structured interview. Data collected on the men’s experiences of recovery were analysed in detail to create a thematic map of their understanding of recovery.

**Results:**

The thematic analysis generated two overarching themes. The first theme focused on psychological recovery in terms of diminished preoccupations with food and disordered eating behaviours, allowing them to eat more freely. They also experienced growth of self-confidence, insight and interpersonal relationships. The second theme viewed recovery as a recursive process without a definite endpoint. The men positioned themselves at different points of the recovery journey and emphasised the importance of support systems and improved resilience to deflect triggers that would otherwise cause relapse.

**Conclusions:**

Recovery was recognised as an improvement in the men’s psychosocial wellbeing from a previous level of functioning. It was a journey which was with no definite endpoint but one that provided benefits such as inner peace, confidence, greater empathy and stronger connections with others around them.

## Plain English Summary

Eating disorders (ED) are very common and affect women more often than men. The aim of therapy in EDs is to reduce distress from problems like binge eating and concerns about body image in order to achieve recovery. However, there is no clear single definition of what constitutes recovery. There is very little research looking at how people who have experienced EDs understand recovery, especially in men with ED. This study therefore looked at how men with EDs understood recovery. After interviewing eight men, we found out that the men were able to comfortably eat food they previously avoided, no longer had behaviours such as over-exercising or excess vomiting and had no issues with their body image. They had become more confident in themselves and gained new personal qualities such as compassion and the ability to relate to others with ED. We also found that men understood recovery as a continuous journey rather than one with a clear ending and avoiding stressful triggers was important to stay in recovery.

## Background

Men comprise a “substantial minority” of people who experience eating disorders (EDs) [1]. Research suggests that they may have different perceptions and experiences of their disordered eating behaviours compared to women [2, 3]. In addition, EDs in men are often underdiagnosed, undertreated and misunderstood [4]. Whilst few people with EDs receive treatment specific to their condition, this problem is even worse for men [5, 6]; in one study only 16% of men with an ED had sought treatment [7, 8]. However, those who do undergo treatment may have a more successful recovery than women. For example, a 2011 Danish cohort study reported that males with EDs had a better outcome than females in terms of body weight restoration and remission of purging behaviours [9]. In Anorexia Nervosa (AN), the remission rates in those with a 5-year history were 39% for females vs. 59% for males. A qualitative analysis of ED symptoms in adolescents reported that males attributed the symptoms arising in the context of fitness, sport and concerns of body images whereas females mentioned family tension as a causative factor [10]. This study also describes that, in comparison to females, males were more cognisant of the effects of an ED and the shame associated with their disordered behaviours may be a motivating factor to engage in therapy which could explain males responding to treatment faster than females [10, 11] .

Whilst recovery from EDs are often seen as the ideal end goal, the meaning of recovery, and what factors contribute to and constitute this meaning of recovery in individuals with ED, regardless of gender, is often poorly understood [12]. The way of defining recovery varies between individuals and across health organisations. In mental health generally, most definitions share overlying ideals about wellbeing and recovery. The Australian Department of Health has outlined six principles for Recovery Oriented Practice in Psychiatry [13]. Their definition of recovery is from the perspective of an individual; where recovery is “to gain and retain hope within themselves, have insight into their capabilities and restrictions, be able to engage in social, occupational and recreational activities, have autonomy and have an identity, purpose and meaning in life”. The focus of this definition is on “internal and external conditions” experienced by individuals in the recovery process. Internal conditions are primarily psychological factors facilitating recovery such as empowerment and connection, whilst external conditions are social facilitators of recovery, including a societal culture promoting positive healing and appropriate services. Similarly, the World Health Organisation’s (WHO) definition of well-being describes a state where “*every individual realizes his or her own potential, can cope with the normal stresses of life, can work productively and fruitfully, and is able to make a contribution to her or his community*.” [14]

In the ED field, the DSM-5 has a more clinical definition of recovery in EDs based on full symptom remission that is sustained for a (subjective) clinician-determined period [15]. A recent systematic review by Bardone-Cone presented research supporting the inclusion of physical, behavioural and psychological/ cognitive criteria for recovery; but only a minor proportion of studies employed all these in their definitions [16]. This review stated that ‘pseudo-recovery’- using only visible indices (weight, behaviours) to measure recovery can create a false sense of hope while the person continues to engage in disordered cognitions (e.g. body image disturbances) thus increasing the risk of relapse. Therefore, understanding psychological/ cognitive recovery can help prognosis in the long-term while physical recovery is only immediate. Several criteria addressing quality of life, comorbid psychiatric illnesses and self-acceptance were proposed to aid in our understanding of recovery. However, this review did not focus solely on one gender or specified such in their paper. Rather, they used a generalised approach to explore recovery and determine a clear definition that constitutes of physical, behavioural and psychological domains of recovery from EDs. Smith et.al. compared the severity of ED psychopathology in males and females using quantitative measures. Their results indicated that females tended to have more severe symptomatic illness than males, but this study failed to discuss to what degree having a less severe psychopathology is beneficial for men or how far it would aid in their recovery process [3].

It is only recently that studies have begun to look into the recovery of men with EDs. It is important to understand how and in what way EDs can have an impact on a man’s life and this includes their understanding of the recovery process and incorporating quantitative and qualitive aspects of positive change. However, to our knowledge there is no similar previous research focusing on men’s experiences of recovery from an ED and what they understand recovery to be. Therefore, this study was designed with the aim to explore how men define/ understand their own ED recovery and what features or aspects of their recovery process constitute their understanding of recovery.

## Methods

### Procedure

The participant recruitment was undertaken by two research students (PT & AC) who placed advertisements across various online platforms. These included the official websites of the Australia & New Zealand Association for Eating Disorders (ANZAED), the Butterfly Foundation, the Centre for Eating and Dieting Disorders, the Australian Clinical Psychology Association (ACPA), the Western Sydney University SONA (student) website and the National Association for Males with an Eating Disorder (NAMED) website. Newspapers and Facebook advertisements were also utilised to aid recruitment.

The respondents to the advertisements who expressed their interest to participate were contacted via email and interviewed by the two research students. The selection criteria of participants ensured that only males aged 18 years and above and those who self-reported being formally diagnosed with an ED and received treatment were eligible to participate.

Ethics: The study was approved by Western Sydney University Human Research Ethics Committee. Protocol number: H11464.

### Participants

Eight men aged 20-33 years (1 unspecified) participated in the study. The age of onset of the ED ranged from 13-19 years. Five men were from the USA, two were Australian, one was from New Zealand. Of the eight men, four reported Anorexia Nervosa, three reported Bulimia Nervosa (BN) and one had Orthorexia. Four men were single, two were partnered, one was married and the other did not specify. In terms of their sexuality, five men reported to be heterosexual while one said he was gay and two men did not specify their sexuality. The various demographic characteristics are summarised in Table 1.

**Table 1:** Participant Demographics

Pseudonym	Age	Country of Origin	Eating Disorder	Marital Status	Other psychiatric comorbidities	Duration of illness
Stevie	33	USA	Bulimia Nervosa	Single	Depression, Anxiety	~ 7 years
Paul	25	USA	Bulimia Nervosa	Single	Depression, Anxiety	5 years
Tom	23	USA	Anorexia Nervosa	Partnered	Depression, Anxiety	5 years
Rony	31	USA	Bulimia Nervosa	Single	Depression Alcohol Use	11 years
Allen	Unspecified	USA	Orthorexia^a^	Unspecified	Anxiety	~ 7 years
Jim	20	Australasia	Anorexia Nervosa	Single	Anxiety	4 years
Harry	31	Australasia	Anorexia Nervosa	Single	Unspecified	Unspecified
Mike	20	Australasia	Anorexia Nervosa	Partnered	Anxiety	3 years

^a^Allen did not wish to provide a DSM-5 diagnosis. He said it was “closest to Orthorexia … at best, this is disordered eating, coupled with a nasty anxiety disorder”. During the interview he discussed severe persistent eating disorder symptoms such as compulsive exercise, weight concern, body image concern, avoiding social eating, previous low weight, and restrictive dieting. These are all symptoms found in DSM-5 eating disorders, but he was also concerned about ‘clean eating’ and thereby chose ‘orthorexia’ as the diagnosis. For example, he had had therapy but whilst helpful, it focussed on body image and he would have preferred it to extend to have a “good relationship with food” which he “regarded as paramount”.

### Assessment and Materials

Participants were asked to read the information sheet provided and subsequently sign the consent form, thereby expressing their voluntary participation. Participants were to be interviewed to saturation using a semi-structured interview format consisting of a combination of open and closed-ended questions. The questions explored the men’s narratives of their lives with an ED and how they negotiated their identities, their treatment experiences and their recovery journeys.

Questions in the interview were developed by the primary researchers (PT, PH, JC, AC) and covered detailed aspects of the participants’ lives from demographics, factors contributing for the development of the EDs, disordered behaviours, thoughts and perceptions, treatment and recovery. Specific questions on recovery included: Do you consider yourself recovered from the eating disorder? If so why?; In terms of recovery what would you say are at the moment?; Can you tell me your story of recovery?; Were there any particular events that stand out in your story of recovery?; Do you have any supports with your eating?; Do you feel now like the same person you were when you were experiencing the eating disorder?; . Do you feel like a different person than before your eating disorder?; How do you think your life has changed with your experience of an eating disorder?; and, Would your life be different if you did not experience an eating disorder? If so how?

Interviews were conducted via telephone or Zoom video conferencing with each interview lasting for about one hour. The interviews were audio-recorded, transcribed and de-identified through use of pseudonyms to protect confidentiality.

### Thematic Analysis

An inductive thematic analysis was performed using the Five-stage Framework method by Pope et al^18^. The first stage was familiarisation of the data and the generation of items using participant phrases, key incidents and participant actions. Using the process of constant comparison in the second stage, items were compared with the dataset to generate emergent analytical categories. Similar data were grouped together while contrasting data were flagged for a later analysis. Categories were added to reflect any information in the transcripts which were perceived to be important by either the participants or analysts. As such, discrete and subtle comments or remarks about a particular topic were assigned a category. By comprehensively sifting through all the categories and by consensus, we were able to arrive at major categories which truly reflected our hypotheses. At the third stage, data were indexed, and the categories refined; transcripts were annotated with numerical codes. The fourth stage was charting. Charting involved re-arranging the data according to the appropriate part of the themes. The fifth and final stage, mapping and interpretation, involved using charts to define concepts and finding associations between themes to provide explanations to the findings.

Data in this project were analysed by two authors: a psychiatrist with an expertise in EDs (PH) and a final year medical student (RLB). The two raters met and discussed and came to a consensus on the final themes, thus differences were resolved by consensus as described by Pope et al. [17].

## Results

The thematic analysis resulted in two main themes and several subthemes. The first theme encompassed concepts of recovery in terms of improved psychological status and the second theme viewed that recovery as a recursive process without a definite endpoint. The participants were at different stages of recovery. Thus, some reflected on being currently recovered whilst others reflected on what this might mean in the future. Saturation was reached on all main themes regarding psychological recovery.

### Theme 1: Psychological Recovery

- Development of better relationships with food and freedom from disordered eating and anxieties about food and body image

In this theme, all men spoke about recovery allowing them to eat freely, eat previously prohibited foods and lose fear and preoccupations surrounding food, disordered eating and body weight. Being recovered from an ED was often described in terms of being released from anxiety and other negative states that existed or remained. Tom spoke about “living without even worrying about it”. Stevie spoke about simply “enjoying putting clothes on”. Allen said he would like a life where he would worry about things that “*actually matter”.* Paul had a positive outlook on different food types and perceived to have a healthy relationship with food. After treatment, Rony “felt pretty good about life”, adding that he “would feel free” without the constant urge to binge, purge or over-exercise.

EXTRACTS 1

**Harry, 31 years, (AN)**: I started to be more open to trying things that the psychologist was suggesting and that really changed my behaviour. Things like challenging my thoughts and also trying out new things like going out and eating things that I avoided eating for a long time.

**Jim, 20 years, (AN)**: admitted that he had no *( … ) fear of or actively engaging in trying to be underweight, not avoiding things...*

b) Personal growth and improved interpersonal relationships as a beneficial effect of recovery

In all participants, where recovery from an ED occurred it was a time of growth in personal self- confidence and regard, and as well as in interpersonal relationships. For two it had led to becoming a therapist and an advocate for other men with EDs. The men described growth in self-efficacy and relationships with others as a bidirectional process. For example, for Allen it would have the practical consequence of being able *to “*ask friends to go out to dinner, instead of staying in with a meal that I know the contents of” and for Rony, pictured as “ … me and my girl lying in bed...I would not go out for a run... I’d just hang out with her.”

The men also engaged in identity negotiations as they narrated their recovery that included a greater connection with themselves through their self-reflected values (extracts 2).

EXTRACTS 2

**Paul, 25 years, (BN)**: I feel like my mind and body are ( … ) centred and I feel like I am a good person ( … ) it’s made me more aware of other people’s emotions, I think I have a deeper sympathy and I am more empathetic towards people … I don’t care if people judge me … .I feel good I feel centred …

**Tom, 23 years, (AN)**: it’s opened up a little bit of awareness, I think. I think that I am much more sensitive to those around me ( … ) it’s definitely opened my eyes to the world in that there are probably more people suffering than I realised*.*

**Jim, 20 years, (AN)**: ( … ) it also taught me a lot about myself and what my strengths are … it’s given me a lot of positive things in my life, a lot of skills and a lot of qualities like being compassionate, being respectful …

Through the ED experience and its recovery these men learnt lessons about themselves and what mattered to them, including a “deeper sympathy” (Paul), increased sensitivity (Tom), respect and compassion (Jim) towards others, including in their suffering (Tom). These self-understandings expanded these men’s vision of themselves whereby they prioritised relationships, which is not as common in men as in women [18]. Furthermore, recovery was experienced by Paul as greater integration between his body and mind (“centred”) and reclaiming the sense of himself as “a good person” that was likely to have been somewhat eroded by the ED experience.

c) Developing insight/understanding of the illness

It was common for the participants to realise the gravity of their plight when they were at their lowest points in life. An awakening of their inner understanding of themselves occurred as they struck ‘rock-bottom’, which contributed to a path to recovery. For example, at the point where he was “very undernourished, very sick”, Mike recounted beginning to “accept that it was an eating disorder”. Paul’s positioning of the ED as unsustainable (“this is not a sustainable way of living”), prompted more active engagement in treatment on his third attempt.

EXTRACTS 3

**Stevie, 33 years, (BN)**: For couple of years I had no insights and then I had a little insight and that’s kind of grown and grown*.*

**Tom, 23 years, (AN)**: There was a part of me that still wanted to live, part of me that still wanted to actually achieve in life, and I think that’s what got me to do it [treatment]*.*

**Jim, 20 years, (AN)**: ( … ) I wanted to do things in life like study and build relationships ( … ) so in order to do those things I realised I needed to work on that [Eating Disorder] …

These men’s unique accounts are built on a sense of hope and “insight” (Stevie) into the ED and its effects on their lives and relationships. For Tom, connection with hope was experienced as “a part of me” that “still wanted to actually achieve in life” and for Jim, as prioritising achievement through his studies and cultivating meaningful relationships with others. These men’s insights highlight that in addition to recognising the seriousness of an ED, they were also faced with their hopes and how the ED risked eroding these.

### Theme 2: Recovery is not clear

- Recovery lacks a definition

Although it was evident that the participants were experiencing some form of recovery from their disordered eating patterns and behaviours, none of them reported to be completely recovered or in remission. Each appeared to be at different points along the recovery process.

EXTRACTS 4

**Mike, 20 years, (AN)**: … I am pretty close to fully recovered so my relationship with food is a lot easier. I don’t have so much of a preoccupation as I used to be with it*.*

**Stevie, 33 years, (BN)**: I think I am a work in progress ( … ) I am not recovered yet ( … ) it has changed over time is a very significant factor …

**Paul, 25 years, (BN):** ( … ) recovery is not a black and white thing so for men that get out, they don’t always have to be, you know be in recovery and it’s not a one-track road, you might relapse, and you might make mistakes but that’s okay. ( … ) I don’t know if I believe that full recovery is possible ( … ) I think you are always in recovery, I don’t think you are ever recovered*.*

These men positioned themselves differently on the notion of recovery that assumes that there is an endpoint to the ED that is recognisable and definable by oneself and/or others [19]. Mike positioned himself as “close to recovery”, Stevie as “a work in progress” whereas Paul eschewed the notion that “full recovery is possible”. Rony used the word “recovering” rather than “recovered” which highlights that recovery is not a static process. In doing so, the majority of these men argued against the assumption of linear progress that builds the dominant clinical view of recovery and in doing so, reconstructed different ways of understanding their recovery as journeys rather than as an endpoint.

b) Unclear endings: the need for ongoing support and development of resiliency

Even after symptomatic recovery, the participants stressed the need for ongoing support – either from a health care professional or family and friends or both. They described a need to be alert to stress and specific ‘triggers’ (Stevie) of lapses. Paul and Rony spoke about their mothers and their girlfriends as key supports. Jim and Tom highlighted the role of access to follow-up treatment sessions to “make sure I’m not falling back”. Mike spoke about the role of *“supportive people in my life … ”* to help maintain the ‘*Positive changes with treatment”.* And he commented that his concerns about his body “*mostly comes up again … at times of stress*”.

An important aspect of the recovery journey was having skills and knowledge to maintain that recovery by self-awareness and knowing how to respond to triggers.

EXTRACTS 5

**Paul, 25 years, (BN)**: ( … ) when you have an eating disorder ( … ) it’s like an actual mental illness that you always have to be aware of, and just aware of what triggers you ( … ). I’ve managed to minimise my triggers, I’ve learnt to cope appropriately in healthy ways ( … )*.*

**Jim, 20 years, (AN)** reflected that he was now “*able to build on my personal strengths”* and would be able to *“counter them”* (triggers/stress) “*later in my life”.*

**Mike, 20 years, (AN)** reported that *“In times of stress or insecurity it will shoot and trigger”.* And that therapy had been helpful in giving him “*useful life skills”.*

**Fig. 1:**
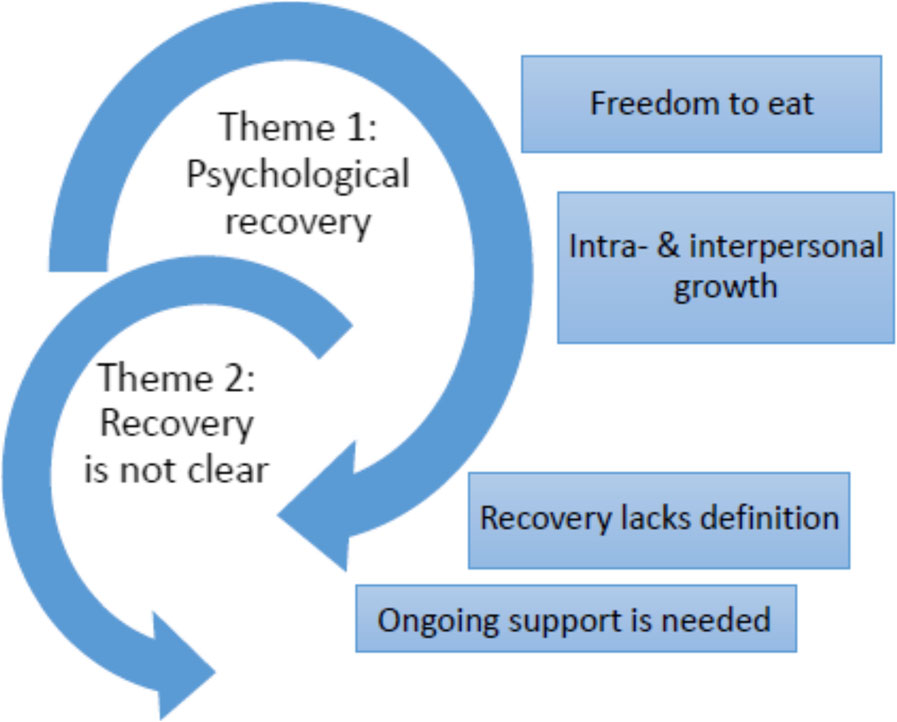
Concept map of thematic analysis: The Journey to Recovery

The development of coping strategies and resilience through therapy was experienced by these men as important in their recovery to maintain change and prevent relapse. This included minimising “my triggers” (Paul), building on “my personal strengths” (Jim) and the development of “useful life skills” (Mike). Implicit in Paul’s narrative is some of the difficulties of navigating a sense of identity with the positioning of his experience as a “mental illness” that is, when does a person diagnosed as disordered become un-disordered and who decides? [20] Furthermore, implicit in Paul and Jim’s use of “my” was the importance of therapy addressing their unique issues and strengths and being focused on insight generation and skills building. The main features of the themes discussed above are summarised in a concept map as displayed in Fig. 1.

## Discussion

In this first qualitative study of men’s understanding of recovery we found two noteworthy themes; the significance of ‘psychological recovery’ and that ‘recovery is not clear’. These two themes indicate how participants negotiated the meanings they ascribed to recovery throughout their recovery journeys, including over the course of their treatment. The first theme focused primarily on the psychological/ cognitive ED recovery through the perspectives of the participants. Over time, ongoing therapy promoted the gradual development of tolerance to certain groups of food that the men previously avoided, and they reported a clear decline in their disordered behaviours such as purging or excessive exercise, but more importantly a reduction in preoccupation with food and body image. The intense sense of control that is a common characteristic quintessential to EDs no longer obstructed their daily lives and gave them a release from their anxiety and other negative states. Recovery was also a period of growth in self-confidence amongst the participants and contributed to the development of interpersonal relationships. Self-awareness of their emotions and that of others grew during the recovery process and as a result of the improved interpersonal relationships, they were able to empathise with people experiencing an ED, especially other men. Through this process, they negotiated a sense of identity built on relational values such as respect and compassion. This is in contrast to the more typical trajectory for young men that risks reduced relational connection to others and the ‘bleak emotional landscape’ that is usually seen in the stoic masculine personality [17].

The concept of freedom from the ED and its restrictions on food, eating and life as expressed by the men in this study has been found in studies of women. For example, a quantitative study of 1052 women in Norway reported that women did not want the ED to control every aspect of their lives and being ‘fed-up’ with living with an ED to be turning points in their journey to recovery [21]. These factors are consistent with those views expressed by our participants as they began to accept the reality of life with an ED which was the primary stepping-stone to recovery. By understanding that EDs can affect anyone, and that it is not just a ‘female disorder’ helped them to move forward as a part of their recovery process and thereby understand what recovery could mean to them. Our findings are also consistent with the concepts expressed in *Recovery Oriented Practice* in mental health [13]. The men experienced internal conditions such as ‘*hope, healing, empowerment and connection*’ and external conditions that facilitated recovery including ‘*implementation of human rights, a positive culture of healing, and recovery-oriented services*’ [13]. This theme supports Bardone-Cone’s systematic review findings that psychological recovery is crucial in assessing outcomes of ED after treatment [16]. Improvement in the overall daily psychosocial functioning of the men suggests an improvement in their quality of life; another factor that is cited and supported in the literature, particularly Bardone-Cone et al. (2018) [16]. The concept of a “turning point “as found in research in women was also strongly mirrored by these men where they expressed engagement in recovery and treatment as a time of gaining understanding and insight [21, 22]. The realisation/ acknowledgement of the negative consequences of an ED led to this “turning point” followed by an elevated “commitment” to recover [21].

In the second theme we explored the ambiguity surrounding the definition of recovery. From analysing the data, it was evident that the men defined recovery to be a period of time devoid of symptoms and preoccupations with food and body image, a definition synonymous with remission. Furthermore, this period of time was highly variable amongst each participant due to the diverse treatments they received. As all the men reported to be ‘recovering’ or ‘in recovery’ as opposed to ‘fully recovered’, this suggests that recovery from ED has no definite endpoint and is rather an ongoing and recursive process. However, they understood that in order to move forward through this process of recovery they must be vigilant in avoiding triggers that would result in a relapse. Recovery was maintained by continuous support from family, friends and therapists and by avoiding triggers that would cause relapse of disordered cognitions and behaviours. There seemed to be a sense of apprehension when the men spoke about their recovery journey; almost as if they were afraid to say they had ‘fully recovered’. The uncertainty of how far the men had recovered and lacking confidence to admit they are free from the disorder conveyed the sensitivity and fragility of this symptom-free time period.

Findings from studies in women are strikingly similar to these issues and the perspective of recovery as an evolving process rather than a static state. In one study, 20 women with a history of AN were interviewed to generate their definitions of recovery [23]. Of the 20 women, 65% defined recovery in terms of resolution of symptoms such as ‘being able to eat comfortably’ and the absence of disordered cognitions. An additional 35% were ‘ambivalent’ about recovery and defined it as an ongoing process while others found it difficult to define recovery. Another 20% defined recovery in a ‘social/ interpersonal’ context where they were able to form close interpersonal relationships with other people and experienced a boost in confidence and self-esteem. Interestingly, 25% of the women did not believe in recovery at all. Ideas defining recovery such as ‘ambivalence about recovery’, improved self-awareness and insight, confidence, development of strong interpersonal relationships and resolution of body image issues were similar across most qualitative studies exploring recovery in women with an emphasis on the equal importance of physical and psychological recovery [20, 24]. However, some studies suggest recovery should be defined more subjectively, claiming that patients should only be labelled as ‘recovered’ if they perceive themselves to be, without the need to satisfy objective criteria for recovery [24]. Taken together, it appears men and women perceive recovery similarly albeit with varying aetiological and treatment factors driving recovery.

Interestingly, the men did not place much emphasis on physical parameters influencing their understanding of recovery such as weight stabilisation and body image. This contrasts with findings in women. For example, Darcy et al. also reported regaining physical health, weight gain and restoration of menses were important aspects defining recovery [22]. Only two men mentioned the improvement in their weight in their recovery narratives. This does not seem to be due to less awareness of body weight or shape, as men have been found to experience more body image issues than women [10]. Rather the men in this study were interested to highlight that recovery extended beyond symptom improvement towards greater self-awareness and intra- and inter-personal connection.

### Strengths and Limitations

The strengths of this study lie in the methodology used. The extensive recruitment process saw advertisements placed widely across several online platforms and newspapers, looking for participants from various backgrounds. This resulted in individuals from countries other than Australia expressing their interests to participate thereby helping us to capture the variations in experiences of men with EDs based on location. Additionally, only applicants who satisfied the strict selection criteria were eligible to participate to ensure standardisation of the study. The two authors (RLB and PH) repeatedly familiarised themselves with the transcribed interviews in order to synthesise as many themes as possible. Furthermore, we were able to reach saturation on all main themes. The limitations are that the primary author (RLB) did not personally interview the participants thereby any potential physical aspects pertaining to recovery that might have been explored further (owing to the author’s medical background and as might have occurred using a licensed provider) did not occur. Additionally, the primary author (RLB) would have been able to follow-up on the initial responses and it would have been interesting to observe whether a male interviewer yielded different versions of the responses in comparison to a female interviewer. It is also possible that thematic analysis with regards to physical health recovery did not reach saturation point. The men who were included in this study came from “Western” societies; recovery in men with EDs from other cultures may have produced different results. There was a lack of other common EDs such as Binge Eating Disorder (BED), Other Specified Feeding or Eating Disorder (OSFED) and Unspecified Feeding or Eating Disorders (UFED) amongst our participants and we recognise this as a limitation on our study. Thus, there is need for triangulation with other samples of men who have experienced these disorders. Whilst homogeneity in samples facilitates investigation of what is common or similar [25] the EDs experienced by the men share symptom profiles and treatments which are increasingly “transdiagnostic” [26]. Further studies should investigate the transferability of these findings to men who experience these and other EDs. The second theme was arrived at due to the men being in various stages of the recovery process; perhaps it would have resulted in the emergence of different themes if the men were closer in their stages of recovery/ further away from illness. Additionally, participants were not invited to review their transcript analysis for member checking- this would have helped to improve the validity of the interviews.

### Implications for future research

There is potential for future research to explore this topic further and support our findings. Triangulation of this study using other population groups could assist in establishing validity and reliability of this study. Such groups could be women with EDs, health professionals who treat individuals with EDs, and men with EDs from diverse cultural backgrounds. A quantitative study could test the frequency of these themes in a larger sample size or determine the quantitative aspects of recovery such as physical parameters and improvement of symptoms. Our findings could inform health professionals treating individuals with EDs, particularly male patients, to tailor their therapy to focus not only on resolution of physical symptoms but also to place an emphasis on psychological aspects of recovery which are important for the long-term prevention of relapse. It is also imperative for health professionals to understand what patients may find as important in their recovery journey; whether it is the development of stronger interpersonal relationships or freedom from disordered cognitions and behaviours.

## Conclusion

The men in this study understood recovery to be an improvement in their psychosocial wellbeing from a previous level of functioning and impairment with little-to-no emphasis on weight and other physical improvements in their health. For these men, recovery was defined as a continuous, on-going journey that was diligently maintained by avoiding triggers that would cause a relapse. This journey included a greater connection within themselves and with others that built on their self-reflected values and hopes for their futures.

## Data Availability

Data are available from Phillipa Hay for collaborative projects and as auspiced by the Western Sydney University Human Research Ethics Committee. The dataset used during the current study is not publicly available due to ethics restrictions and need to maintain participant anonymity.

## Abbreviations

ACPA: Australian Clinical Psychology Association
AN: Anorexia Nervosa
ANZAED: Australia & New Zealand Association for Eating Disorders
BED: Binge Eating Disorder
BN: Bulimia Nervosa
DSM-5: Diagnostic and Statistical Manual of Mental Disorders, Fifth Edition
ED: Eating Disorder
NAMED: National Association for Males with an Eating Disorder
OSFED: Other Specified Feeding and Eating Disorders
UFED: Unspecified Feeding and Eating Disorders
WHO: World Health Organisation
